# Alterations in PD-L1^+^ Myeloid Cells and Immune Infiltration Are Associated with Atezolizumab and Paclitaxel Therapy Success in a Triple-Negative Breast Cancer Model

**DOI:** 10.3390/medicina62030600

**Published:** 2026-03-22

**Authors:** Kerim Bora Yilmaz, Ece Tavukcuoglu, Hamdullah Yanik, Pelin Seçken, Ertugrul Celik, Sumeyra Guler, Mehmet Mert Hidiroglu, Ibrahim Burak Bahcecioglu, Ismail Erturk, Mehmet Ali Gulcelik, Derya Karakoc, Gunes Esendagli

**Affiliations:** 1 Department of Medical and Surgical Research, Institute of Health Sciences, Hacettepe University, 06800 Ankara, Turkey; 2 Department of General Surgery, Gulhane Training and Research Hospital, University of Health Sciences, 06010 Ankara, Turkey; 3 Department of Basic Oncology, Cancer Institute, Hacettepe University, 06800 Ankara, Turkey; 4 Department of Pathology, Gulhane Training and Research Hospital, University of Health Sciences, 06010 Ankara, Turkey; 5 Department of Surgical Oncology, Gulhane Training and Research Hospital, University of Health Sciences, 06010 Ankara, Turkey; 6 Department of Medical Oncology, Gulhane Training and Research Hospital, University of Health Sciences, 06010 Ankara, Turkey; 7 Department of General Surgery, Faculty of Medicine, Hacettepe University, 06800 Ankara, Turkey

**Keywords:** triple-negative breast cancer, immune checkpoint inhibitors, atezolizumab, paclitaxel, combination therapy, tumor immune microenvironment, metastasis, treatment response

## Abstract

*Background and Objectives:* A combination of chemotherapy and immunotherapy may improve cancer treatment outcomes; however, determining which patient groups will benefit from immunotherapy is critical. Triple-negative breast cancer (TNBC) achieves limited benefit from immune checkpoint inhibitors (ICIs) and anti-PD-L1 blockade therapy. *Materials and Methods:* In this study, PD-L1 expression levels in myeloid-derived cells in the tumor microenvironment were determined in an experimental TNBC model. *Results:* Compared with tumor cells, granulocytes, monocytes, and macrophages had significantly higher PD-L1 expression. CD206^+^ tumor-associated macrophages (TAMs) expressed the highest level of PD-L1. PD-L1 positivity in TAMs was also significantly high in the lung, liver, lymph node, and spleen. Despite treatment initiation in late-stage tumorigenesis, the combination of paclitaxel and the anti-PD-L1 monoclonal antibody atezolizumab significantly reduced tumor growth. In addition, lung metastasis burden was reduced with combined treatment compared with chemotherapy or anti-PD-L1 monotherapy alone. *Conclusions:* As a result, alterations in PD-L1^+^ myeloid cells and immune infiltration can be associated with atezolizumab and paclitaxel therapy success in triple-negative breast cancer.

## 1. Introduction

Triple-negative breast cancer (TNBC) accounts for 10–20% of all breast cancer cases. It is characterized by the absence of an estrogen receptor (ER), a progesterone receptor (PR), and a human epidermal growth factor receptor 2 (HER2) [1]. TNBC is the most aggressive subtype of breast cancer and has a high risk of metastasis [2]. Owing to the absence of hormone receptors and HER2 expression, TNBC lacks eligibility for conventional hormone-based or HER2-targeted therapies, and systemic treatment has historically relied on chemotherapy despite recent biomarker-driven advances. Standard treatments for TNBC include surgery, chemotherapy, and radiotherapy [3].

As part of first-line neoadjuvant therapy for TNBC, paclitaxel (PCX) is a key chemotherapy agent, but its effectiveness remains limited [4]. TNBC exhibits higher immunogenicity compared with other subtypes of breast cancer because of high tumor mutational burden and immune cell infiltration [5]. Therefore, immunotherapeutic strategies have become a significant advancement in TNBC. Anti-PD-L1 (Atezolizumab) was the first immunotherapy for TNBC approved by the U.S. Food and Drug Administration [6]. PD-L1 can be highly expressed by other cells, especially tumor-associated macrophages in the tumor microenvironment and TNBC tumor cells. Thus, the blockade of PD-L1 by other cells in the tumor microenvironment can elicit strong anti-tumor immune responses even if the tumor cells lack PD-L1 expression [7]. However, the success of immunotherapy alone has been limited, and its combination with chemotherapy agents has emerged as a promising approach to improve immunotherapy effectiveness in TNBC [8]. The 4T1 murine TNBC model was selected for its aggressive growth, spontaneous metastatic capacity, and well-documented ability to induce systemic expansion of tumor-associated myeloid cells, including MDSCs. This feature makes it particularly suitable for studying PD-L1 dynamics within the myeloid compartment of the tumor microenvironment.

In this study, we aimed to evaluate the effectiveness of PCX and anti-PD-L1 combination therapy using an experimental triple-negative breast cancer model.

## 2. Materials and Methods

### 2.1. Tumor Model

The 4T1 breast cancer cell line (American Type Culture Collection LGC, Promochem, Wesel, Germany) was cultured in RPMI 1640 medium (Biological Industries, Kibbutz Beit Haemek, Israel) supplemented with 10% FBS (Biological Industries) and 1% penicillin–streptomycin (Biological Industries) in a humidified atmosphere with 5% CO_2_ at 37 °C. Female BALB/c mice (6–8 weeks old; Hacettepe University Laboratory Animals Research and Application Center, Ankara, Türkiye) were subcutaneously injected in the right flank with 4T1 cells (2.5 × 10^4^ cells/50 µL).

### 2.2. Paclitaxel and αPD-L1 Treatments

When the geometric mean of the tumors reached approximately 0.6 cm (on day 14), the mice were divided into four groups: control, paclitaxel (PCX), αPD-L1 (atezolizumab), and PCX + αPD-L1. Each experimental group consisted of six mice (*n* = 6 per group; total *n* = 24). Every 2 and 3 days, PCX (final concentration 20 mg/kg) and αPD-L1 (final concentration 10 mg/kg), respectively, were injected intraperitoneally. The control group was intraperitoneally injected with a saline solution as a control. Treatments lasted for 1 week, and at the end of the treatment period, the mice were sacrificed.

### 2.3. Cell Isolation and Flow Cytometry

After sacrifice, tumor, lung, liver, spleen, and lymph nodes were collected from each animal in each experimental group. An average of six animals were utilized per experimental group; however, the number of samples obtained from distinct tissue compartments ranged between 3 and 8 due to independent repetitions in the experiments. Statistical significance was determined by employing appropriate analyses tailored to the respective sample sizes. Flow cytometric analyses were performed using samples obtained from each animal. The organs were mechanically and enzymatically processed in a solution containing collagenase type II (100 U/mL, Nordmark, Uetersen, Germany) and DNase I (200 U/mL, Sigma-Aldrich, St. Louis, MO, USA) for 2 h. Then, cells were filtered through a 40 µm strainer to obtain a single-cell suspension. The cells were labeled with anti-mouse CD45 (30-F11), CD11b (M1/70), F4/80 (BM8), CD206 (C068C2), Ly6G (1A8), Ly6C (HK1.4), and PD-L1 (10F.9G2; BioLegend, San Diego, CA, USA). Following the incubation period with the antibodies, the cells were washed and analyzed by flow cytometry (FACSCanto II, BD, San Jose, CA, USA). Percentages and median fluorescent intensities (MFIs) were determined according to the autofluorescence control. The gating strategy for flow cytometry given in Supplementary Figure S1.

### 2.4. Histopathological Analysis

Tumor and lung samples were collected from all animals in each experimental group. The samples were fixed in 10% formalin and embedded in paraffin. After hematoxylin and eosin staining, histopathological analysis was performed under a light microscope. Percentages of tumor necrosis and lung metastasis densities were calculated. Representative images are shown in the figures.

### 2.5. Statistical Analysis

Statistical analysis was performed using one-way ANOVA followed by Tukey’s post hoc test. Data are presented as mean ± SEM. A *p*-value < 0.05 was considered statistically significant.

## 3. Results

### 3.1. PD-L1 Expression Is More Prominent on Tumor-Infiltrating Myeloid Cells in the TNBC Model

**Figure 1:**
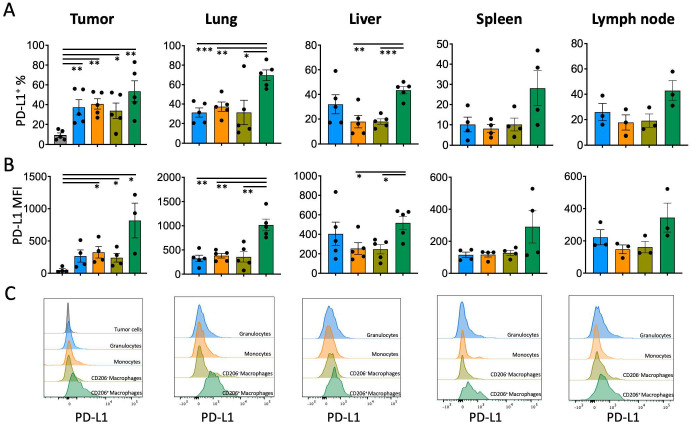
Analysis of PD-L1 expression on tumor cells and tumor-infiltrating immune cells. (**A**) Proportions of PD-L1^+^ cells, (**B**) PD-L1 MFI, and (**C**) representative flow cytometry histograms of PD-L1 expression. Data are presented as mean ± SEM (* *p* < 0.05, ** *p* < 0.01, *** *p* < 0.001). Statistical significance was determined by one-way ANOVA. Each black dots represent an indiviual mouse.

In clinical practice, PD-L1 expression on tumor cells is used as a decision parameter for PD-L1 or PD-1 immune checkpoint inhibition (ICI) therapy, but PD-L1 may also be highly expressed on the surface of immune cells infiltrating the tumor microenvironment [9]. Given that myeloid cells can better communicate with PD-1^+^ T lymphocytes, the PD-L1 levels of these cells may be critical for ICI therapy responses. To test this hypothesis, a TNBC tumor model without PD-L1 expression was established, and PD-L1 levels of tumor-infiltrating monocytes, granulocytes, macrophages, and CD206^+^ tumor-associated macrophages (TAMs) were determined. All tested myeloid cells expressed the PD-L1 molecule significantly more than tumor cells and cells of non-immune origin (CD45-negative) (PD-L1^+^ % on tumor cells 9.2 ± 1.1, on granulocytes 37.3 ± 7.7, on monocytes 40.7 ± 5.2, on CD206^−^ macrophages 33.8 ± 7.8, on CD206^+^ macrophages 53.5 ± 10.6). The highest level of PD-L1 expression was on CD206^+^ TAMs (Figure 1A–C). The PD-L1 expression on myeloid cells populating the lung and liver, which are the main metastasis target organs for 4T1 TNBC cells, was also significantly higher in CD206^+^ macrophages. There was also a significant trend of PD-L1 increase on CD206^+^ macrophage surfaces found in immune organs such as lymph nodes and the spleen (Figure 1A–C).

### 3.2. Combination of Paclitaxel Chemotherapy with Anti-PD-L1 Blockade Hinders Tumor Growth and Metastasis

**Figure 2:**
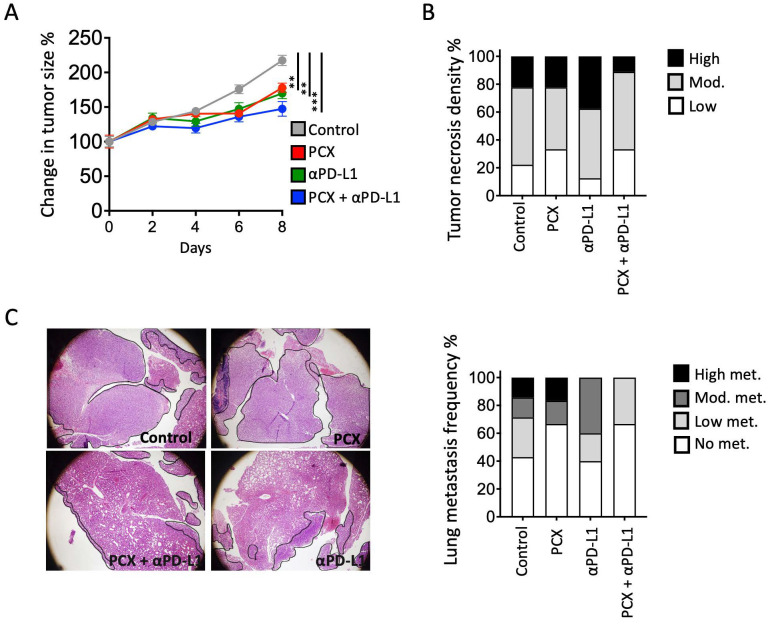
(**A**) Change in tumor size under paclitaxel, anti-PD-L1, and combination treatments, (**B**) proportions of tumor necrosis, and (**C**) representative hematoxylin and eosin (H&E)-stained lung tissue section and lung metastasis frequency. Data are presented as mean ± SEM (** *p* < 0.01, *** *p* < 0.001). (Black outlines indicate metastatic tumor regions in lung parenchyma.) Statistical significance was determined by one-way ANOVA. *n* = 6 per group.

Next, mice with TNBC were treated with paclitaxel chemotherapy, anti-PD-L1 (Atezolizumab) ICI immunotherapy, and a combination of both. Although tumor growth was impeded in all three groups compared with the control group, a significant decrease in tumor size was observed after the combined therapy (Figure 2A). Consistent with the reduction in tumor size, fewer necrotic areas in the tumors of the combined chemotherapy and ICI group were observed (Figure 2B). Most importantly, the combination of these two treatments significantly reduced lung metastasis (Figure 2C). High metastasis burden was observed in mice treated with paclitaxel alone. Although metastasis burden decreased in mice treated with anti-PD-L1 blockade therapy alone, the frequency of animals with metastasis was not significantly different. The combination of chemotherapy and anti-PD-L1 immunotherapy treatments significantly reduced both metastasis burden and frequency in the lung.

### 3.3. Paclitaxel and Atezolizumab Synergistically Remodel the Tumor Immune Microenvironment in 4T1 Tumors

Both chemotherapy and immune checkpoint inhibitors are now widely recognized for their combined potential to improve anti-tumor treatment efficacy [10]. In that manner, paclitaxel eradicates tumors by inducing immunologic cell death and enhancing tumor immunogenicity [11]. In combination with anti-PD-L1 inhibitors, it may further increase the anti-tumor immune response. To assess how this combined therapy affects the immune composition of the tumor microenvironment and other tissues, flow cytometric analyses were performed in the 4T1 murine breast cancer model. Our results demonstrated distinct alterations in immune cell composition and activation states, providing mechanistic insights into the therapeutic efficacy of this combination approach. Specifically, tumor CD45^+^ immune cells increased after anti-PD-L1 treatment compared with paclitaxel chemotherapy and combined therapy. Although not correlated with treatment efficacy, the increased number of cells in the tumor with monotherapy can be interpreted as immune cell migration to the tumor site. For the lung, paclitaxel treatment reduced immune cell infiltration compared with the control group (control group 33.2 ± 1.4 × 10^3^ cells/mg, paclitaxel-treated group 24.7 ± 2.8 × 10^3^ cells/mg). This decrease could be due to conventional chemotherapy, which has an immunosuppressive effect on secondary lymphoid organs, or its cytotoxic effects, which affect not only cancer cells but also dividing immune cells. So, it might dampen immunity during treatment. Immunosuppression can be a significant limitation for chemotherapy efficacy, especially when combined with immunotherapies.

**Figure 3:**
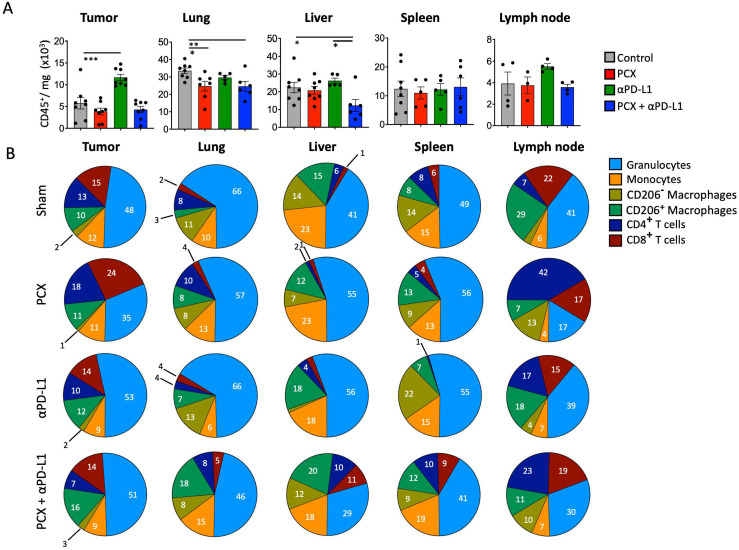
(**A**) Amount of CD45^+^/mg under paclitaxel, anti-PD-L1, and combination treatments at tumor, lung, liver, spleen, lymph node. Data corresponding to each individual tumor-bearing mouse are presented as black dot plots to illustrate the heterogeneity of the obtained results. (**B**) To facilitate the tracking of mean immune cell fluctuations across experimental groups and organ compartments, the average values for each group were represented as pie charts. Percentage distribution of immune cells (granulocytes, monocytes, CD206^−^ macrophages, CD206^+^ macrophages, CD4^+^ T cells, and CD8^+^ T cells) under paclitaxel, anti-PD-L1, and combination treatments. Data are presented as mean ± SEM (* *p* < 0.05, ** *p* < 0.01, *** *p* < 0.001). Statistical significance was determined by one-way ANOVA.

In addition, combined treatment in TNBC significantly decreased immune cell infiltration in the liver compared with the control group (c) (Figure 3A).

Subsequently, the frequencies of different immune cells in tissues were analyzed in the TNBC mouse model. It was observed that although paclitaxel treatment increased CD8^+^ T cells, the frequency of granulocytes decreased in the tumor. This finding suggests that chemotherapy promotes anti-tumor immunity by increasing the number of cytotoxic T cells at the tumor site. In the lung, it was observed that granulocytes decreased with paclitaxel treatment in one of the metastatic TNBC sites compared with the control group. This decrease in granulocytes with paclitaxel alone and combination therapy reduced neutrophil infiltration at the site, which might block metastatic niche formation. Granulocytes also decreased with combination treatment compared with the control group, and the frequency of CD206^+^ macrophages increased.

In the liver, the frequency of granulocytes, monocytes, and CD206^+^ macrophages decreased in the combination therapy group compared with the control group. This finding suggests that combination treatment reduces the frequency of myeloid cell infiltration in the liver, potentially limiting the establishment of a metastatic microenvironment. The liver is particularly important because it serves as a major extravasation site for circulating tumor cells; therefore, reducing myeloid cell infiltration may impede metastatic progression. In the spleen, although the total number of immune cells did not change, the frequency of CD4^+^ and CD8^+^ T cells was significantly lower with anti-PD-L1 therapy compared with other treatment groups. This finding could be due to T cell redistribution, as they may have migrated from the spleen to peripheral tissues like tumors or metastatic sites.

**Figure 4:**
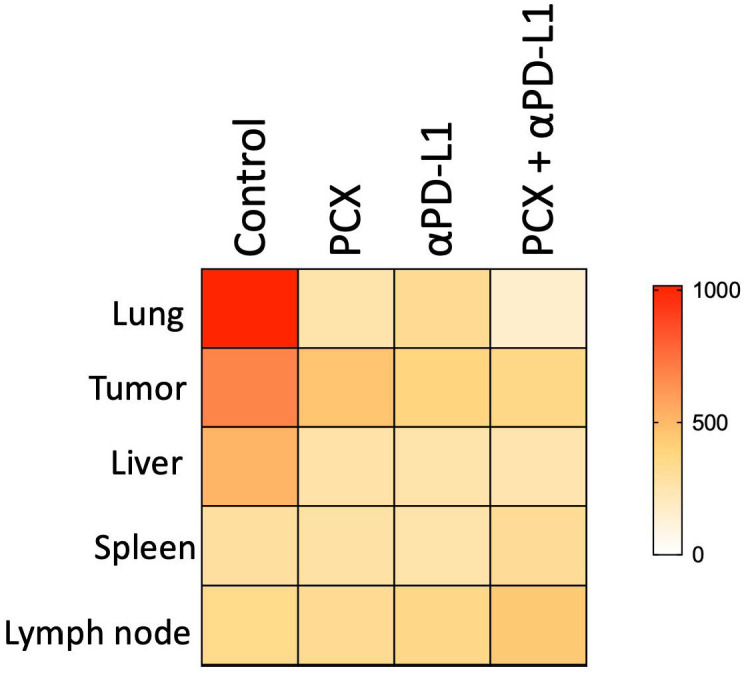
Change in PD-L1 MFI on 206^+^ M2 macrophages in different tissues (lung, tumor, liver, spleen, lymph node) under paclitaxel, anti-PD-L1, and combination treatments. Data are expressed as mean ± SEM. Statistical significance was determined by one-way ANOVA.

Finally, when examining the lymph nodes, CD4^+^ T cells were significantly increased in the paclitaxel treatment group compared with the other groups, while granulocytes were less frequent. This increase in the CD4^+^ T cell population suggests that paclitaxel treatment activates and expands T cells at tumor sites. CD4 T cells are crucial for coordinating the anti-tumor immune response, so paclitaxel may improve adaptive immune activation. On the other hand, the significant decrease in granulocytes in this compartment suggests reduced suppression by myeloid cells infiltrating the microenvironment (Figure 3B). Since PD-L1 expression on CD206^+^ macrophages is higher, MFI (mean fluorescent intensity) values were used to generate a heat map of different compartments in TNBC mice under different treatments and their combinations. The tissue-wide reduction in PD-L1 expression on CD206^+^ macrophages in mice suggests that treatments target immunosuppressive cells in vivo. These decreases across multiple tissues indicate a more systemic effect rather than a purely local change, suggesting a broader impact of the treatment on the immunosuppressive myeloid compartment in tumor-bearing mice. The biological significance of this observation in the mouse model lies in the functional role of PD-L1 on macrophage surfaces. PD-L1, or programmed death ligand 1, is a key immune checkpoint molecule that delivers inhibitory signals to T cells expressing its receptor PD-1, thereby suppressing anti-tumor immune responses. PD-L1 contributes to the creation of an immunosuppressive microenvironment by actively dampening T cell activation, proliferation, and effector functions. Therefore, the treatment-induced decrease in PD-L1 MFI on CD206^+^ macrophages in our mouse model is thought to be mechanistically associated with the removal of or substantial reduction in the immunosuppressive environment within the tissues. These findings provide important preclinical evidence that treatment can target checkpoint ligand expression on immunosuppressive myeloid cells (Figure 4).

**Figure 5:**
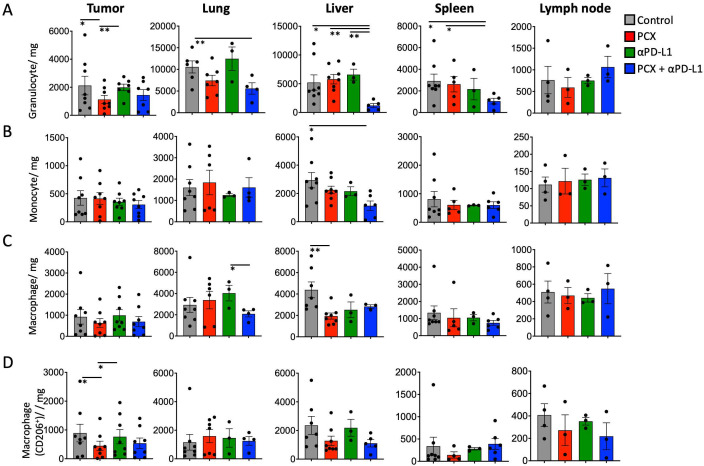
(**A**) Amount of Granulocytes/mg, (**B**) monocytes/mg, (**C**) macrophages/mg, (**D**) macrophages CD206^+^/mg under paclitaxel, anti-PD-L1, and combination treatments at tumor, lung, liver, spleen, and lymph node. Data corresponding to each individual tumor-bearing mouse are presented as dot plots to illustrate the heterogeneity of the obtained results. Data are presented as mean ± SEM (* *p* < 0.05, ** *p* < 0.01). Statistical significance was determined by one-way ANOVA.

To better understand the immunological mechanisms underlying the different treatment responses, myeloid cell populations (cells per gram of tissue) within different tissue compartments were examined, given the critical role of myeloid cells in the tumor microenvironment and in regulating anti-tumor immunity. In primary tumors, the myeloid cell population can be changed by treatment. In our results, granulocyte infiltration was significantly reduced by paclitaxel monotherapy compared with the control group. This effect may be attributable to the ability of chemotherapy to deplete or prevent cell recruitment into the tumor microenvironment. On the other hand, anti-PD-L1 monotherapy increased the number of granulocytes in the tumor microenvironment, suggesting that checkpoint blocking without chemotherapy-induced cell death and immunologic signals may enhance myeloid cell migration to the tumor microenvironment. After paclitaxel treatment, there was a significant decrease in CD206^+^ macrophages in tumors. On the other hand, anti-PD-L1 monotherapy led to a significant increase in the frequency of CD206^+^ macrophages in tumors (Figure 5C,D).

**Figure 6:**
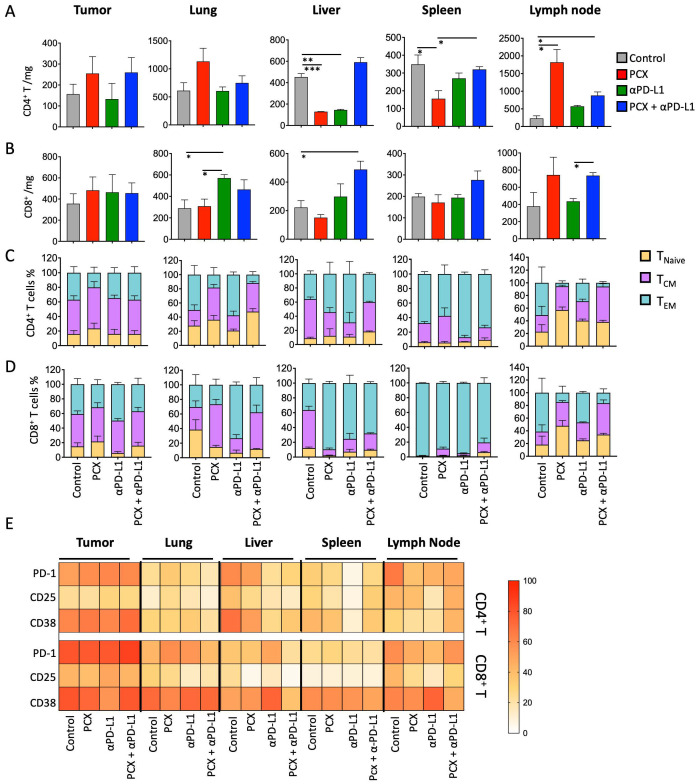
(**A**) Amount of CD4^+^/mg and (**B**) CD8^+^/mg under paclitaxel, anti-PD-L1, and combination treatments at tumor, lung, liver, spleen, and lymph node. (**C**) CD4^+^ T cell distribution (T naïve, T_CM_: central memory T cell, T_EM_: effector memory T cell). (**D**) CD8^+^ T cell distribution (T naïve, T_CM_: central memory T cell, T_EM_: effector memory T cell). (**E**) Analysis of T cell activation markers (PD-1, CD25, CD38) under paclitaxel, anti-PD-L1, and combination treatments at tumor, lung, liver, spleen, and lymph node. Data are presented as mean ± SEM (* *p* < 0.05, ** *p* < 0.01, *** *p* < 0.001). Statistical significance was determined by one-way ANOVA.

After analyzing myeloid cell populations in different compartments in the TNBC mouse model under various treatment conditions, we examined how T cell numbers and functional properties change in the tumor and other compartments. When the number of CD4^+^ T cells was examined, it was observed that both paclitaxel and anti-PD-L1 treatments decreased the number of CD4^+^ T cells compared with the control group, and no significant change was observed in the combination treatment group. In the spleen, paclitaxel treatment significantly decreased the number of CD4^+^ T cells compared with the control and combined treatment groups (control group: 348.9 ± 52.5 cells/mg; paclitaxel-treated group: 155.6 ± 45.2 cells/mg). In the lymph node, paclitaxel and combination treatment increased the number of CD4^+^ T cells compared with the control group (control group: 230 ± 73.5 cells/mg; paclitaxel-treated group: 1820 ± 361.8 cells/mg; combination treatment group: 880 ± 100 cells/mg) (Figure 6A). In addition, when the number of CD8^+^ T cells was examined, it was found that anti-PD-L1 therapy significantly increased CD8^+^ cell counts in the lungs compared with the paclitaxel and control groups, whereas combination therapy significantly increased T cell infiltration in the liver compared with the control group (Figure 6B).

After comparing frequencies according to T cell activation status, CD4^+^ T cell subtypes were similar across the different treatments. However, in the lung, it was observed that T_EM_ cells converted to T_CM_ cells after paclitaxel and combination therapy. In the liver, the number of T_CM_ cells decreased after paclitaxel and combination therapy, whereas T_EM_ cells increased in distribution. In the lymph nodes, the number of CD4^+^ TEM cells decreased in all treatment groups compared with the control group. Notably, anti-PD-L1 therapy was associated with a more pronounced alteration in CD4^+^ TEM cell distribution compared with the other treatment groups. In the lung, naïve CD8^+^ T cells decreased under all treatment conditions except the control group. The number of CD8^+^ T_CM_ cells increased with paclitaxel and combination therapy. When subsets of CD8^+^ T cells in the liver were examined, the number of naïve CD8^+^ T cells decreased, and T_EM_ cells increased across all administered treatments. In addition, in the spleen, CD8^+^ TCM cells increased significantly compared with the control group after treatment. In the lymph nodes, T_EM_ CD8 cells decreased in the control group, and T_CM_ cells increased (Figure 6D).

After examining the T cell subsets, T cell activation-related surface markers were examined, and their percentage distributions were analyzed in the tumor and various organs under different treatments using a heatmap. When activation markers (PD-1, CD25, CD38) on CD4^+^ and CD8^+^ T cells in the tumor were examined, it was observed that CD38 expression was decreased in CD8^+^ T cells with anti-PD-L1 treatment. When activation markers were examined in the lung, PD-1 expression was increased with paclitaxel and anti-PD-L1 therapy compared with the control group. In the liver, PD-1, CD25, and CD38 expression levels were significantly decreased. In the spleen, PD-1, CD25, and CD38 expression in CD4^+^ T cells was reduced following anti-PD-L1 therapy compared with the control and other treatment groups. Finally, analysis of the lymph nodes revealed that PD-1 expression was reduced across all treatment groups compared with the control group. Moreover, CD25 and CD38 expression levels were decreased following paclitaxel and anti-PD-L1 therapy (Figure 6E).

## 4. Discussion

To date, immunotherapy has emerged as a promising strategy in cancer treatment due to its favorable safety and success in highly immunogenic tumors [12]. However, tumors characterized by active suppression of anti-tumor immunity, immune cell subversion, elevated levels of anti-inflammatory mediators, and a non-inflamed phenotype lacking cytotoxic T-lymphocyte infiltration often exhibit limited responsiveness to immunotherapy [13]. Tumor mutational burden also plays a critical role in immune recognition and therapeutic response.

TNBC is a subtype of breast cancer that is theoretically expected to respond to immunotherapy due to its relatively high mutation load and immune cell infiltration [5]. Nevertheless, TNBC patients generally derive limited benefit from conventional targeted therapies compared with other breast cancer subtypes [4]. The distinct extracellular matrix composition and mesenchymal characteristics of the TNBC tumor microenvironment promote chronic inflammatory signaling [14]. However, this inflammation frequently drives tumor-supportive macrophage polarization and regulatory T cell responses [15].

Our findings demonstrate that tumor-associated macrophages (TAMs), particularly CD206^+^ macrophages, represent major sources of PD-L1 expression within the TNBC microenvironment. Importantly, PD-L1 expression was observed not only within the primary tumor but also in metastatic target organs and secondary lymphoid tissues, suggesting that PD-L1 assessment restricted to tumor cells may underestimate the overall immunosuppressive burden in TNBC.

As expected, although the efficacy of paclitaxel chemotherapy in TNBC was limited, anti-PD-L1 treatment showed some anti-tumor efficacy. This is generally consistent with human clinical studies reported in the literature [16,17,18]. The combination of paclitaxel chemotherapy and anti-PD-L1 was determined to be the best combination for slowing tumor growth. In addition, the reduction in metastasis burden indicates that PD-L1 expression is important not only on tumor cells but also on myeloid cell surfaces in the tumor microenvironment, and even on myeloid cell surfaces, such as macrophages, in the structures of other organs. Our data provide a mechanistic explanation for clinical observations in which PD-L1-negative TNBC tumors still derive benefit from immune checkpoint inhibition, implicating PD-L1-expressing myeloid cells as critical regulators of therapeutic response.

Combination paclitaxel and atezolizumab treatment in TNBC causes a selective redistribution of the immune response, prioritizing local immunity over systemic immune response. The decrease in immune cells in the spleen and the increase in CD8^+^ T cell frequency show that paclitaxel enhances the anti-tumor T cell response when checkpoint blockade increases their functional activity [19]. In addition, both monotherapy and combination therapy suppressed granulocyte infiltration at the lung site, potentially preventing metastatic niche formation through reduced neutrophil-derived immunosuppression. Moreover, the increase in CD206^+^ macrophages under combination therapy reflects polarization toward a pro-inflammatory phenotype due to atezolizumab-induced interferon signaling [20]. The reduction in PD-L1-expressing myeloid cells in metastatic target organs suggests that combination therapy may disrupt the formation or maintenance of a pre-metastatic niche in TNBC. These findings indicate that both paclitaxel and atezolizumab synergistically enhance local anti-tumor immunity while suppressing immunosuppressive myeloid cells at both primary and metastatic sites.

The trafficking of T cell populations across anatomical compartments and tumors reveals distinct immunological mechanisms underpinning monotherapy versus combination treatment in triple-negative breast cancer. Both paclitaxel and anti-PD-L1 monotherapies reduced tumor-infiltrating CD4^+^ T cells, suggesting that initial chemotherapy and checkpoint blockade might shift CD4^+^ T cell distribution away from tumor tissue. However, combination therapy maintained CD4^+^ T cell numbers compared with the control group, indicating that CD4^+^ T cells are required for effective anti-tumor immunity [21,22]. In addition, paclitaxel significantly reduced splenic CD4^+^ T cells, suggesting mobilization from secondary lymphoid organs to peripheral tissues, while there was an increase in CD4^+^ T cells in tumor-draining lymph nodes. This mobilization suggests that paclitaxel promotes priming and activation of CD4^+^ T cells within lymph nodes while recruiting them to primary tumor tissues [23,24]. Anti-PD-L1 monotherapy enhanced CD8^+^ T cell infiltration in the lung, the major metastasis site for the TNBC model, suggesting that checkpoint blockade may drive these cells to metastatic sites [25]. In contrast, combination therapy uniquely promoted significant CD8^+^ T cell infiltration in the liver, indicating that the addition of paclitaxel redirects cytotoxic T cell trafficking toward an alternative metastatic site. This tissue-specific migration of CD8^+^ T cells likely indicates chemotherapy-induced changes in adhesion molecules and chemokine gradients toward the tumor that occur with T cell activation due to PD-L1 blockade. The compartment-specific patterns of T cell activation show the different immunological effects of combination versus monotherapy in TNBC. In the tumor microenvironment, decreased CD38 expression on CD8^+^ T cells under anti-PD-L1 treatment suggests a change toward a functional, classically activated phenotype [26]. Increased PD-1 expression in the lung under both paclitaxel and anti-PD-L1 treatment suggests repeated antigen presentation at metastatic sites, whereas significant reductions in PD-1, CD25, and CD38 in the liver with combination therapy indicate an organ-specific immune response [27]. Anti-PD-L1 monotherapy specifically suppressed CD4^+^ T cell activation markers (PD-1, CD25, CD38) in the spleen, suggesting selective suppression on T cell activation [28]. Within tumor-draining lymph nodes, uniform PD-1 reduction across all the treatments confirmed successful checkpoint blockade, while additional CD25 and CD38 decreases with combination therapy indicated efficient T cell activation. Finally, these parameters show that paclitaxel and anti-PD-L1 combination therapy generate an organized immune response with activation in secondary lymphoid organs and differential T cell states at primary and metastatic sites that enhance anti-tumor immunity [29,30].

This study has several limitations. The findings are based on a murine TNBC model, and extrapolation to human disease should be made with caution. In addition, functional assays to directly assess macrophage polarization and T cell effector function were not performed. Future studies incorporating human samples and functional validation will be necessary to confirm the translational relevance of these findings.

## 5. Conclusions

Collectively, our findings demonstrate that the therapeutic efficacy of paclitaxel and anti-PD-L1 combination therapy in TNBC is mediated not only by tumor-intrinsic effects but also by extensive remodeling of myeloid and T cell compartments across primary and metastatic sites. Targeting immunosuppressive myeloid PD-L1 may represent a key mechanism underlying effective immune checkpoint inhibition in TNBC.

## Data Availability

Data are available upon reasonable request.

## Supplementary Materials

The following supporting information can be downloaded at: https://www.mdpi.com/article/10.3390/medicina62030600/s1, Figure S1. Gating strategy for flow cytometry is shown.

## Abbreviations

The following abbreviations are used in this manuscript:

TNBC	Triple-negative breast cancer
ICI	Immune checkpoint inhibitors
PD-L1	Programmed death ligand 1
TAMs	Tumor-associated macrophages
PCX	Paclitaxel (chemotherapy group)
ER	Estrogen receptor
PR	Progesterone receptor
HER2	Human epidermal growth factor receptor 2
FDA	Food and Drug Administration
MFI	Mean fluorescence intensity
H&E	Hematoxylin and eosin
SEM	Standard error of the mean
FBS	Fetal bovine serum
RPMI	Roswell Park Memorial Institute medium
BALB/c	Bagg albino laboratory-bred strain c (mouse strain)
CD	Cluster of differentiation
TCM	Central memory T cell
TEM	Effector memory T cell
αPD-L1	Anti-PD-L1 monoclonal antibody
