# Supplementary figures and images for: Alterations in PD-L1+ Myeloid Cells and Immune Infiltration Are Associated with Atezolizumab and Paclitaxel Therapy Success in a Triple-Negative Breast Cancer Model
Source: Medicina (Kaunas). 2026 Mar 22;62(3):600. doi: 10.3390/medicina62030600 (PMC13028344; doi:10.3390/medicina62030600)

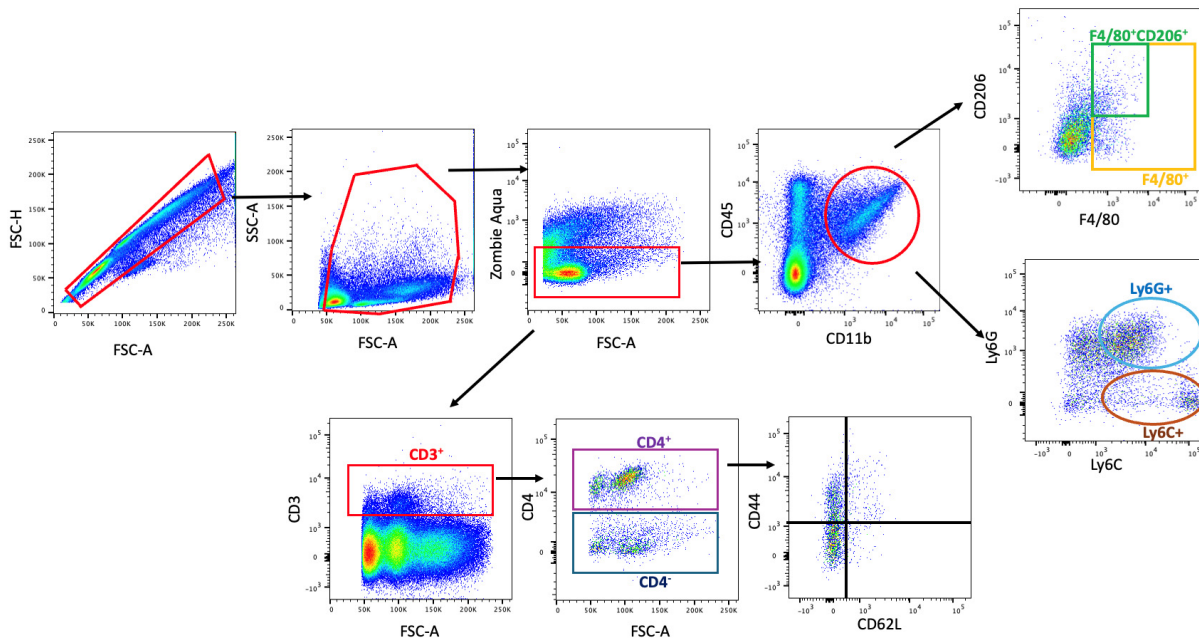

**Supplementary Figure S1.** Gating strategy for flow cytometry is shown.

Supplement: Supplementary file 1 [file medicina-62-00600-s001.zip › medicina-4144225-supplementary.pdf]
